# Successful perinatal management in pregnant women with R9 limb-girdle muscular dystrophy and left ventricular ejection fraction of 27%: a case report

**DOI:** 10.3389/fmed.2025.1576161

**Published:** 2025-08-08

**Authors:** Wenchi Xie, Lok Chong Huang, Dan Luo, Landan Kang, Koubihan Hu, Xiong Liu, Jie Mei

**Affiliations:** ^1^Department of Obstetrics and Gynecology, The Affiliated Hospital of Southwest Medical University, Luzhou, Sichuan, China; ^2^Kenneth P. Dietrich School of Arts and Sciences, University of Pittsburgh, Pittsburgh, PA, United States; ^3^College of Clinical Medicine, University of Electronic Science and Technology of China, Chengdu, China; ^4^Department of Obstetrics and Gynaecology, Sichuan Provincial People's Hospital, University of Electronic Science and Technology of China, Chengdu, China

**Keywords:** case report, limb-girdle muscular dystrophy, pregnancy, cardiomyopathy, heart failure

## Abstract

A 29-year-old woman with muscle weakness at 26 + 5 weeks of pregnancy was hospitalized for heart failure, and genetic testing revealed that she had R9 limb-girdle muscular dystrophy (LGMDR9). Since LGMDR9 is associated with cardiac and respiratory dysfunction, we promoted fetal lung maturation and performed an emergency cesarean section at 29 + 2 weeks to prevent further deterioration of cardiac function and muscle weakness. This case highlights the importance of choosing the right mode of delivery, timing of delivery, and early genetic testing for LGMDR9 patients.

## Introduction

Heart failure (HF, LVEF<30%, NYHA functional class III–IV) is classified as WHO category IV (contraindication to pregnancy), and its risk associated with maternal death or severe morbidity is extremely high (1). Although there have been case reports of patients with HF becoming pregnant and successfully delivering, multidisciplinary treatment is needed for such patients, especially for those with genetic disorders that lead to progressive cardiac decompensation (2).

The fukutin-associated protein-encoding gene (FKRP) encodes a protein required for the posttranslational glycosylation of *α*-dystroglycan, which is essential for membrane stability. Mutations in FKRP have been shown to cause R9 limb-girdle muscular dystrophy (LGMDR9, formerly known as LGMD2I), an autosomal recessive muscular dystrophy characterized by progressive weakness and atrophy of proximal muscles, variable age of onset, normal cognitive function, elevated serum creatine kinase (CK), and frequent cardiac and respiratory dysfunction (3–5). LGMD is very rare, with an incidence of 0.43 per 100,000 people (6). Pregnancy rates in patients with LGMDR9 are similar to those in the general population, but muscle weakness worsens in approximately half of the pregnant women. In addition, pregnant women with LGMDR9 have increased perinatal mortality due to respiratory and circulatory complications and weak smooth muscle contraction during labor (7, 8).

With the advent of genetic testing, an increase in the number of LGMDR9 cases is being detected. Therefore, there may also be a greater need for physicians skilled in managing LGMDR9 in pregnant women. Currently, research on pregnancy management in LGMDR9-related diseases is limited. Although there have been reports of similar cases [e.g., (6)], no personalized pregnancy management framework has been provided for LGMDR9-affected pregnant women. This study, by combining clinical indicators and pregnancy risk assessments, proposes an initial individualized risk assessment framework that can serve as a reference for managing similar cases in the future.

## Case report

**Figure 1:**
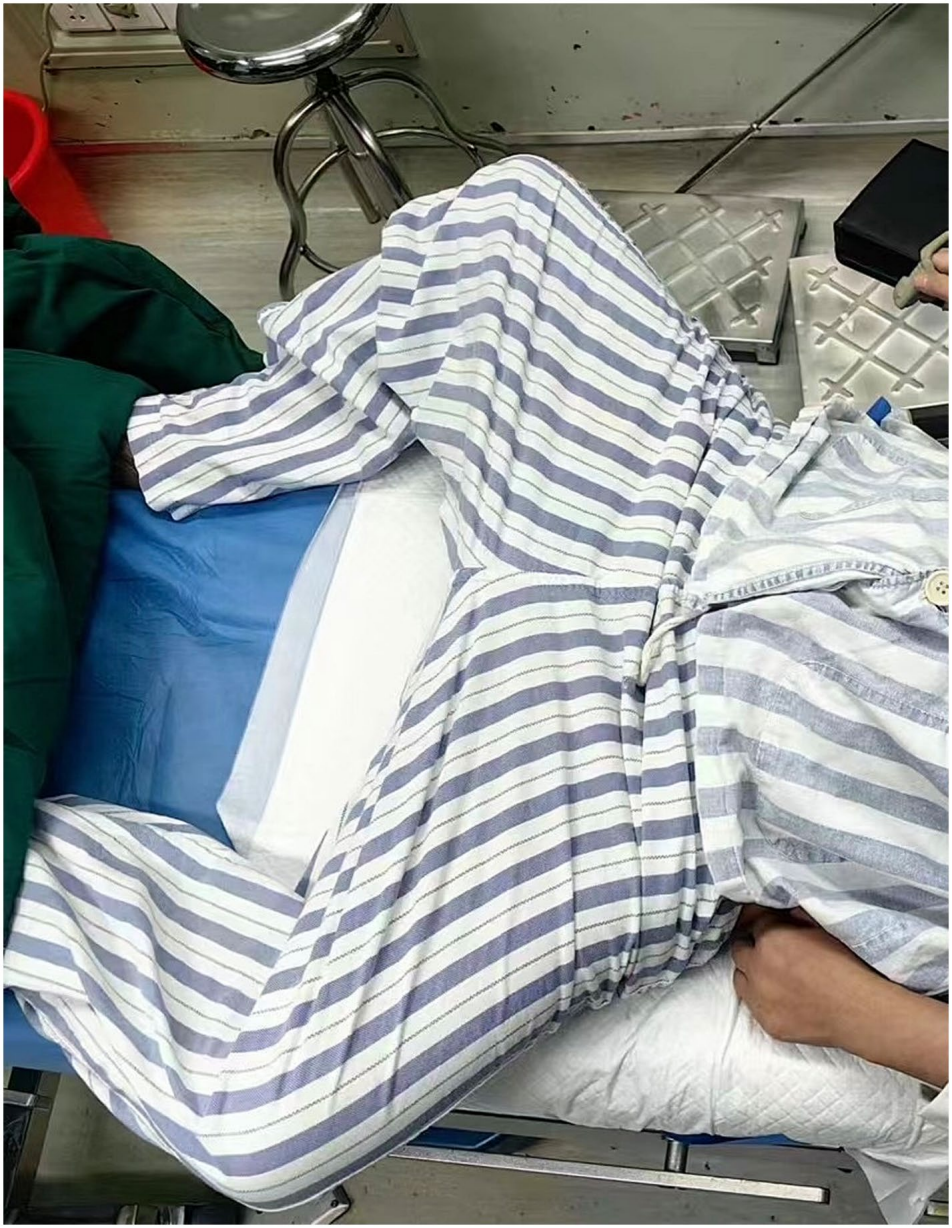
Patient’s pelvis is affected; the lower limbs cannot be extended in the supine position, but are rather abducted and externally rotated.

A 29-year-old woman at 26 + 5 weeks of pregnancy was referred to our hospital in January because of abnormal cardiac function after pneumonia treatment. The main symptoms at presentation were dyspnea, obvious exertion after activity, and occasional chest pain. She had a 21-year history of muscle weakness prior to this pregnancy. Ultrasound revealed a broadly reduced amplitude of motion in the left ventricular wall, overall uncoordinated movement, and an LVEF of 27%. Other parameters of cardiac function were as follows: BNP, 55.4 pg./mL; creatine kinase, 154 U/L; ECG, sinus rhythm; and the electrical axis was normal. Notably, although the patient’s LVEF was significantly reduced, her BNP level remained relatively low. This may be due to the early stages of cardiac dysfunction after pneumonia. In addition, her young age, limited activity due to longstanding muscle weakness, and reduced cardiac preload may have contributed to the lower BNP level. The patient had a history of myasthenia for 21 years, no genetic testing was performed, and the pregnant woman’s parents were close relatives. Chest examination revealed a uniform heart rhythm, and no murmurs were heard. Head extensor muscle strength was grade 0. Proximal muscle weakness was evident: In the upper extremities, proximal strength was grade 3 on the right side and grade 2 + on the left side; in the lower extremities, muscle strength was grade 2. Distal muscle strength was relatively preserved. Spinal deformity and Achilles tendon contracture were also observed (Figure 1).

After patient admission, we developed a diagnosis and treatment plan through multidisciplinary consultation. Muscular dystrophy and muscular dystrophic cardiomyopathy were considered. To confirm the diagnosis, electromyography revealed that the examined muscles were prone to chronic myogenic damage and that the serum creatine kinase level was 154 U/L. High-throughput sequencing was used to detect whole-exome genes, and one homozygous mutation in the FKRP gene was analyzed: nucleotide 823 changed from cytosine C to thymine T (c.823C > T), resulting in a change from arginine to cysteine (p. Arg275Cys) (Table 1). According to ACMG guidelines^1^, this variant was preliminarily determined to be pathogenic (PP3 moderate+PM3 very strong+PM2 supporting). According to the patient’s history, she had normal cognitive function and had experienced slowly progressive proximal limb weakness since the age of 8 years. However, a definitive diagnosis was not established until after the patient’s admission, when neurologists suspected LGMDR9 based on the patient’s symptoms and medical history and initiated genetic testing, which ultimately confirmed the diagnosis.

**Table 1:** Results of gene sequencing.

Gene	*Chromosomal position*	*Exons*	*Nucleotide amino acids*	*Zygosity*	*Normal frequency*	*Prognosis*	*ACMG pathogenicity analysis (score)*
*FKRP*	chr19:472 59530*	NM-0243 01.5; exon4	c.823C > T (P. Arg275 Cys)	hom	0.0001945	D	Pathogenic

* Associated with muscular dystrophy, limb girdle 2I: Lin, et al. Brain Dev, 29, 234, 2007 [PMID: 17055682] |30,293,248|30,816,495|32,576,226|34,602,496 ClinVar: Conflicting classifications of pathogenicity, Walker–Warburg congenital muscular dystrophy | Muscular dystrophy–dystroglycanopathy (congenital with brain and eye anomalies), type A5|not provided | Autosomal recessive limb-girdle muscular dystrophy type 2I.

Considering the patient’s impaired limb muscle strength and reduced cardiac pumping function (LVEF of 27%), and based on the New York Heart Association (NYHA) classification, the patient’s heart function has progressed to NYHA class III, further indicating the deterioration of cardiac function. With increasing gestational age, the amount of blood returning to the heart increases, and malignant arrhythmia, cardiac arrest, and sudden cardiac death may occur at any time. After two courses of dexamethasone sodium phosphate to promote fetal lung maturation, we terminated the pregnancy via cesarean section under general anesthesia at 29 + 2 weeks. General anesthesia was chosen over regional techniques due to the patient’s neuromuscular condition—specifically, severe proximal muscle weakness, spinal deformity, and a high risk of respiratory compromise—allowing for better airway control and reducing the risk of intraoperative respiratory failure. The surgery was successful. She gave birth to a healthy boy weighing 980 grams with APGAR scores of 6, 9, and 10 at 1, 5, and 10 min, respectively. Considering the weakness of uterine smooth muscle contraction, a uterine band suture was used to stop bleeding.

After delivery, the patient was transferred to the intensive care unit (ICU), and the newborn was admitted to the neonatal intensive care unit (NICU). During the ICU stay, initial blood gas analysis showed pH 7.39, pO₂ 26.3 kPa, pCO₂ 6.3 kPa, HCO₃⁻ 28.5 mmol/L, and lactate 0.5 mmol/L, with blood potassium at 3.3 mmol/L, sodium at 134 mmol/L, and free calcium at 1.12 mmol/L. The patient received targeted treatments, including amlodipine for vasodilation and blood pressure control, opioid sedation to reduce sympathetic stimulation and decrease cardiac workload, levosimendan as a calcium sensitizer to enhance myocardial contractility without increasing oxygen consumption, furosemide for diuresis to alleviate pulmonary congestion, and cefazolin sodium for postoperative infection prevention. Additionally, bronchodilators were used to relieve dyspnea caused by heart failure, and potassium chloride was administered to correct hypokalemia and prevent life-threatening arrhythmias induced by the use of cardiotonic agents and diuretics.

During treatment, continuous monitoring of vital signs and key laboratory parameters was performed. Follow-up blood gas analysis showed an improvement, with pH rising to 7.45, pCO₂ decreasing to 4.5 kPa, and lactate slightly increasing to 0.7 mmol/L, indicating improved oxygenation and acid-base balance. Blood potassium increased to 4.3 mmol/L, blood sodium remained stable at 133–134 mmol/L, and free calcium stayed within 1.11–1.18 mmol/L. On the day following extubation, the patient’s hemodynamic and respiratory status stabilized, and she was transferred back to the general ward. Eventually, both the mother and the newborn were discharged in stable condition without significant sequelae.

After discharge, the patient was prescribed long-term management for persistent heart failure, which included perindopril (ACEI), metoprolol (*β*-blocker), spironolactone (aldosterone antagonist), empagliflozin (SGLT2 inhibitor), coenzyme Q10 (mitochondrial enhancer), and digoxin (positive inotrope). At the same time, genetic testing was performed for the neonate; fortunately, the genetic testing results for the newborn showed a carrier of the FKRP c.823C > T heterozygous mutation, consistent with the carrier status. No homozygous or compound heterozygous pathogenic variants were identified. Two months after discharge, the patient had a follow-up BNP of 48 pg./mL and was in good condition.

## Discussion

Women with LGMDR9 who get pregnant may encounter many challenges. First, muscle weakness symptoms may develop significantly during pregnancy. It manifests mainly as marked weakness in the pelvic girdle and trunk muscles. The contraction of the abdominal or pelvic muscles is important for daily activities, and if not functioning properly, the risk of dystocia increases greatly (9). Second, severe cases of LGMDR9 may be associated with impaired circulatory function, which is further exacerbated by the physiological demands of pregnancy, which may lead to inadequate blood supply to the placenta, fetal growth restriction, and even intrauterine fetal death. In addition, it may lead to the deterioration of maternal cardiac function, which greatly increases perinatal mortality (6). In addition, as the fetus grows and the uterus pushes on the diaphragm, the patient’s ability to breathe may decrease. This may necessitate an early cesarean section. Therefore, patients with muscular dystrophy may have a higher rate of cesarean section (10).

Therefore, early diagnosis is particularly important. A general lack of awareness of LGMD disease among physicians, combined with often vague and non-specific symptoms, makes disease identification and diagnosis difficult. In such patients, their history is first thoroughly examined to determine whether there is a family history of the disease and to understand the onset of symptoms and the distribution of muscle involvement. In recent years, genetic testing has become more widely used for final biochemical confirmation of LGMD because of its higher specificity than muscle biopsy and immunostaining (11). For such patients, genetic testing to confirm the diagnosis of the disease, early exercise training to increase physical fitness, and prevention of infection and heart failure during pregnancy are all recommended (12).

With respect to the mode of delivery, some patients with mild symptoms may try having a vaginal delivery, but they are more likely to have assisted labor and induced labor. In a cohort study, the proportions of patients with LGMDR9 who had assisted labor (35%) and induced labor (70%) were significantly greater than the national average (3 and 27%, respectively) (8). A cesarean section is usually recommended if there are blood gas abnormalities during pregnancy, a lung capacity of less than 1 to 1.5 liters, pulmonary hypertension or HF, diaphragm or abdominal weakness, or pelvic abnormalities. Therefore, for LGMDR9 patients with HF, the cesarean section can be selected to ensure the safety of the mother and child (13). Early cesarean section can shorten the volume loading period and enable a more aggressive approach to HF; however, iatrogenic preterm labor may have a negative impact on the offspring. On the other hand, patients with heart failure are more likely to experience fetal death and intrauterine growth retardation, so we should be more proactive in promoting fetal lung maturation and choosing the right time for cesarean section to terminate pregnancy (14).

In light of the management experience in this case, we propose a preliminary individualized perinatal management framework for pregnant patients with LGMDR9. Its core lies in early risk stratification based on cardiac function, respiratory reserve, and the extent of musculoskeletal involvement. For cardiac assessment, echocardiography and heart failure biomarkers (such as BNP or NT-proBNP) should be performed routinely in early pregnancy and monitored throughout gestation. Particular attention must be paid to left ventricular ejection fraction (LVEF): If LVEF falls below 45%, surveillance should be intensified; when LVEF declines to below 35%, it indicates compromised maternal cardiac reserve and merits evaluation for potential preterm delivery; and if LVEF drops below 30% accompanied by significant symptoms (e.g., dyspnea, chest pain, or fatigue), then elective cesarean delivery should be planned as soon as fetal lung maturation is achieved to prevent malignant arrhythmias, sudden cardiac arrest, or other life-threatening complications. In our case, the patient’s LVEF reached 27%, and after corticosteroid administration, the cesarean section was performed at 29 + 2 weeks, effectively averting further cardiac decompensation.

Respiratory function should be assessed through pulmonary function tests and arterial blood-gas analyses, especially in late pregnancy when diaphragmatic elevation may compromise ventilation. A forced vital capacity (FVC) of less than 50% of predicted or an arterial PaCO₂ above 6.0 kPa should be considered relative indications for early delivery. Neurological evaluation is also critical for anesthetic planning: In patients with pronounced axial or bulbar muscle weakness, regional anesthesia is not recommended; instead, general anesthesia with secured airway control should be preferred.

Furthermore, a multidisciplinary team approach is essential. Obstetricians should lead in coordination with cardiology, anesthesiology, neurology, and neonatology to develop and continuously adjust individualized treatment and delivery plans. All therapeutic decisions—including medication regimens and timing of delivery—must be dynamically tailored to the patient’s evolving clinical status. Postpartum care should be structured, with cardiac function reassessed 6–12 weeks after delivery, and rehabilitation interventions initiated to support long-term muscle function recovery and improve quality of life.

Moreover, planning preconception is important for LGMDR9 patients. Genetic counseling and prenatal diagnosis are desirable, given that they could reduce the birth rate of fetuses with genetic predispositions. LGMDR9 patients with HF can consider the use of *β*-receptor blockers, diuretics, and vasodilators to improve cardiac function before and during pregnancy. Suppression of the renin–angiotensin–aldosterone system should be restored as soon as possible after delivery (2). In LGMDR9 patients with a high risk of sudden cardiac death, ICD implantation can be considered prior to pregnancy. ICD use during pregnancy does not increase the risk of ICD-related complications. If an emergency arises, ICDs are recommended (12).

There are some limitations to consider. The absence of multimodal diagnostic evidence, such as muscle MRI and histopathology (biopsy), is a notable gap, as these tests could offer significant supplementary value in diagnosing LGMDR9. Additionally, carrier screening was conducted only on the newborn, and a more comprehensive family pedigree analysis was not performed. Future studies incorporating these diagnostic methods and broader familial screening would enhance the understanding and confirmation of LGMDR9.

## Conclusion

In this case involving an LGMDR9 patient with HF, we made a genetic diagnosis in time based on the patient’s past medical history and clinical manifestations. We chose the cesarean section to terminate the pregnancy, considering the symptoms of severe cardiac insufficiency, which avoided adverse outcomes. The findings of this case should be used as a reference for the treatment of pregnant women with other genetic diseases.

## Data Availability

The original contributions presented in the study are included in the article/supplementary material, further inquiries can be directed to the corresponding author.

## Footnotes

^1^ http://acmg.cbgc.org.cn
